# Physical activity, BMI and cancer prevention in urban adults

**DOI:** 10.1186/1753-6561-6-S3-P69

**Published:** 2012-06-27

**Authors:** Sally N Akarolo-Anthony, Clement A Adebamowo

**Affiliations:** 1 Nutrition Department, Harvard School of Public Health, Boston, MA, 02215, USA; 2 Epidemiology and Public Health, School of Medicine, University of Maryland, Baltimore, MD, 021201, USA

## Background

Developing countries are undergoing epidemiological transitions with increasing prevalence of Non-Communicable Diseases (NCD) particularly cancers. In many African countries, poor lifestyle choices including dietary patterns, lack of physical activity and weight gain contribute to the risk factors of many cancers. In this study, we evaluated physical activity, carbohydrate intake and anthropometric measurements in an urbanized Nigerian population. Our objective was to measure the prevalent lifestyle, as well as develop and disseminate adequate educational strategies based on our findings, to alleviate the problem in the population.

## Methods

1059 workers at a government office in Nigeria answered an interviewed administered questionnaire and had anthropometric characteristics measured in a study of body size, dietary energy intake and physical activity in 2010.

## Results

In our study population, the mean age (SD) of participants was 41.5 (9.3) years and mean BMI (SD) was 27.0 (4.9). 36% are overweight and 25% are obese. 58.9% reported that they don’t exercise, 11.2% exercise 1day/week, 8.7% exercise 2days/week and 21.2% exercise at least 3days/week. 55% spend at least 6 hours/week sitting at home watching TV, 51% spend at least 6 hours/week sitting doing other things. >90% don’t spend any time on sports (running, biking, tennis, squash, soccer, swimming, hiking, aerobics, and weights). 24% spend >10hours/week walking or standing at home, while 67% spend >10hours/week working at home.

## Conclusion

Our result shows urbanized Nigerian adults adopt a sedentary lifestyle and are more physically active while executing housework than while away from home. As obesity and physical inactivity are modifiable risk factors for NCD, adults should be encouraged to engage in culturally acceptable lifestyle activities that will prevent obesity and promote physical activity as a modality to reduce NCD’s especially cancer incidence in Africa.

